# CD1d levels in peripheral blood of patients with acute myeloid leukemia and acute lymphoblastic leukemia

**DOI:** 10.3892/ol.2014.2208

**Published:** 2014-06-02

**Authors:** WENJIAN GUO, AISHU DONG, CHAO XING, XIAOJI LIN, XIAHUI PAN, YING LIN, BAOLING ZHU, MUQING HE, RONG-XING YAO

**Affiliations:** Department of Medicine, The Second Affiliated Hospital and Yuying Children’s Hospital of Wenzhou Medical University, Wenzhou, Zhejiang 0577, P.R. China

**Keywords:** primary acute leukemia, CD1d, monocytes, CD3^+^CD56^+^ T lymphocytes

## Abstract

The antitumor effect of natural killer T cells has been reported in several studies analyzing the expression of CD1d on antigen-presenting cells (APCs). Therefore, the present study questioned whether APCs may be abnormal in the peripheral blood (PB) of acute leukemia (AL) patients, particularly the levels of CD1d. To improve the understanding of the role of CD1d on APCs, the levels of CD1d on monocytes were analyzed in healthy controls, AL patients and AL patients with complete remission (CR). In addition, the correlation between the number of CD3^+^CD56^+^ T lymphocytes and levels of CD1d on monocytes was analyzed. Flow cytometry was used to determine the levels of CD1d on monocytes and lymphocytes. A significant decrease was observed in the levels of CD1d on monocytes in the PB of acute myeloid leukemia (AML) and acute lymphoblastic leukemia (ALL) patients compared with the healthy controls. Simultaneously, significantly different levels of CD1d on monocytes were identified between the CR-AML and the CR-ALL patients; the levels of CD1d on monocytes remained low in the CR-AML patients, while the levels of CD1d on monocytes recovered in the CR-ALL patients. A significantly negative correlation was observed between the number of CD3^+^CD56^+^ T lymphocytes and the levels of CD1d on monocytes in AL patients. However, a significantly positive correlation was identified between the cytotoxicity of the CD3^+^CD56^+^ T lymphocytes and the levels of CD1d on monocytes. These results suggested that the significantly low levels of CD1d on monocytes may contribute to AML and ALL progression. In addition, a significant correlation was observed between the levels of CD1d on monocytes and the number/cytotoxicity of CD3^+^CD56^+^ T lymphocytes in AML and ALL patients.

## Introduction

Immune evasion is an important mechanism in cancer progression. Several studies have identified abnormal cellular immunity in cancer patients, thus indicating potential new strategies for cancer treatment (1). However, successful immunotherapy requires an improved understanding of the changes in the immune system of cancer patients.

Acute leukemia (AL) is a malignant tumor of the hematological system, characterized by malignant clones of leukemia cells in the bone marrow (BM). AL can be classified as acute myeloid leukemia (AML) or acute lymphoblastic leukemia (ALL), according to the French-American-British system (2). Chemotherapy and stem cell transplantation (SCT) represent the main treatments for AL. Long-term survival may require SCT, which is based on the rebuilding of the immune system to produce a graft-versus-leukemia effect (3). However, the high cost and the significant side effects and mortality limit the applicability of BM transplantations in China. Therefore, novel immunotherapies are currently used and investigation into the abnormalities of the immune system in AL patients has become a hot topic. Several studies have shown changes in the number and function of T-lymphocyte subsets in chronic lymphocytic leukemia (4–7).

CD3^+^CD56^+^ T lymphocytes were first described as a distinct subset of T cells more than one decade ago (8,9). This subset expresses surface receptors that are also found on conventional T cells (such as CD3), together with receptors characteristic of natural killer (NK) cells (including CD56) and, therefore, are also referred to as NKT cells. These cells have already been shown to exhibit antitumor cytotoxicity (10,11); however, unlike classical T cells, which recognize peptides presented by highly polymorphic major histocompatibility complex (MHC) molecules, NKT cells recognize glycolipids via MHC-like, non-polymorphic CD1d molecules (12–21). CD1d-restricted T-cell populations have a physiological role in tumor immunosurveillance, which is mediated at least partly through the maturation of antigen-presenting cells (APCs; including monocytes, macrophages and dendritic cells) and IL-12 induction via NK and CD8^+^ T cells (22–30). In addition, immunity against a number of tumor models has been observed with the therapeutic activation of NKT by selective agonist α-galactosylceramide presented by CD1d^+^ APCs (22–24).

As a number of studies have shown that cancer progression may be associated with the dysfunction of abnormal APCs (31–34), APCs may be abnormal in AL patients. In our previous study, the number and cytotoxicity of CD3^+^CD56^+^ T lymphocytes were found to change in AL patients. In particular, the cytotoxicity of CD3^+^CD56^+^ T lymphocytes was decreased in AL patients. Therefore, we questioned whether APCs may be abnormal in AL patients, particularly the levels of CD1d. At present, no studies have examined the levels of CD1d on the monocytes and lymphocytes in the peripheral blood (PB) of AL patients. The present study compared the levels of CD1d on the monocytes and lymphocytes in patients with primary AL and healthy controls, as well as in AL patients who had achieved complete remission (CR) following chemotherapy. Simultaneously, the correlation between the number of CD3^+^CD56^+^ T lymphocytes and levels of CD1d was analyzed.

## Materials and methods

### Patients

Fresh PB samples were collected from 56 randomly selected patients with primary AL (32 AML and 24 ALL, with the exclusion of acute promyelocytic leukemia) and 28 CR-AL patients (14 AML and 14 ALL, with the exclusion of acute promyelocytic leukemia) who visited the Department of Hematology at the Second Affiliated Hospital of Wenzhou Medical College (Wenzhou, China) for treatment. In total, 18 AML and 10 ALL patients exhibited increased (>10×10^9^/l) white blood cell (WBC) counts at the time of diagnosis (termed AML-1 and ALL-1, respectively), and 14 AML and 14 ALL patients exhibited low (<10×10^9^/l) WBC counts at diagnosis (termed AML-2 and ALL-2, respectively). The patient characteristics are shown in Table I. Normal fresh PB samples were obtained from 20 healthy volunteers. No individuals in the control group were administered any medication or suffered from any known acute or chronic disease. All patients and volunteers provided written informed consent to participate in the study. The study was approved by the ethics committee of the Second Affiliated Hospital & Yuying Children’s Hospital of Wenzhou Medical University (Wenzhou, China).

### Reagents

Monoclonal allophycocyanin mouse anti-human CD45, PerCP-Cy5.5 mouse anti-human CD3, phycoerythrin (PE) mouse anti-human CD1d and fluorescein isothiocyanate (FITC) mouse anti-human CD56 were purchased from eBioscience (San Diego, CA, USA).

### Lymphocyte membrane phenotype and expression of CD1d on monocytes and lymphocytes

Triple-labeling experiments were performed using EDTA-anticoagulated PB samples (AL, CR-AL and healthy controls). Aliquots of 100 μl were incubated for 30 min at room temperature with pretitered dilutions of allophycocyanin-, FITC-, PE- and PerCP-Cy5.5-conjugated monoclonal antibodies against CD45 (HI30), CD3 (OKT3; clone 25; IgG1) and CD56 (IgG1), respectively. Isotype-matched control antibodies conjugated with FITC, PE and PerCP-Cy5.5 were included to establish background fluorescence. Erythrocytes were subsequently lysed by adding 3 ml of NH_4_Cl for 10 min at room temperature. The cells were then washed in phosphate-buffered saline supplemented with 0.1 mM EDTA and 0.02% NaN_2_, and kept on ice until flow cytometric examination.

### Flow cytometry

A flow cytometer (FACSCalibur; BD Biosciences, San Jose, CA, USA) was used for data acquisition and FlowJo software (TreeStar Inc., Ashland, OR, USA) was used for analysis.

### Statistical analysis

Data are presented as the mean ± standard deviation. The significant differences were analyzed by one-way analysis of variance. The correlation between the number of CD3^+^CD56^+^ T lymphocytes and levels of CD1d was analyzed by Pearson’s correlation analysis. P<0.05 was considered to indicate a statistically significant difference.

## Results

### Levels of CD1d in the PB of patients presenting with AL

**Figure 1:**
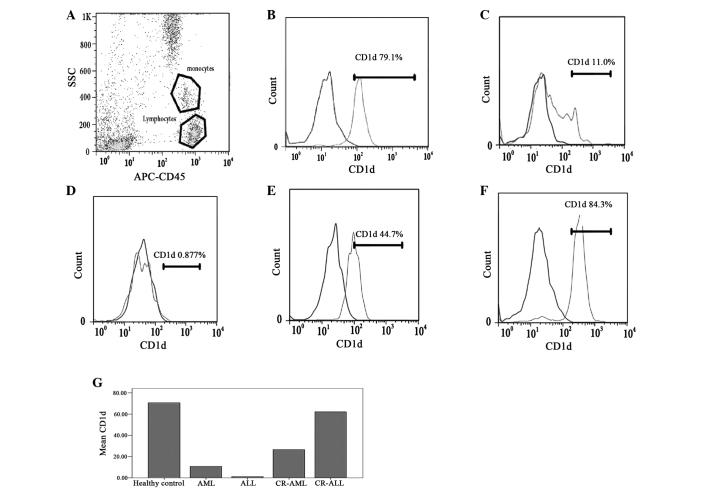
Levels of CD1d on the monocytes in the PB of patients with AL and CR-AL, and healthy controls. The levels of CD1d on the monocytes in the PB of AL patients (AML and ALL) were lower than those in the healthy controls. The change in the levels of CD1d on the monocytes was different in CR-AML and CR-ALL patients. The levels of CD1d on the monocytes remained low in CR-AML patients, but the levels of CD1d on the monocytes recovered in CR-ALL patients. (A) Monocytes and lymphocytes; (B) healthy control, (C) AML, (D) ALL, (E) CR-AML and (F) CR-ALL groups; and (G) the levels of CD1d in the different groups. PB, peripheral blood; AL, acute leukemia; CR, complete remission; AML, acute myeloid leukemia; ALL, acute lymphoblastic leukemia; APC, antigen-presenting cell; SCC, side scatter.

**Figure 2:**
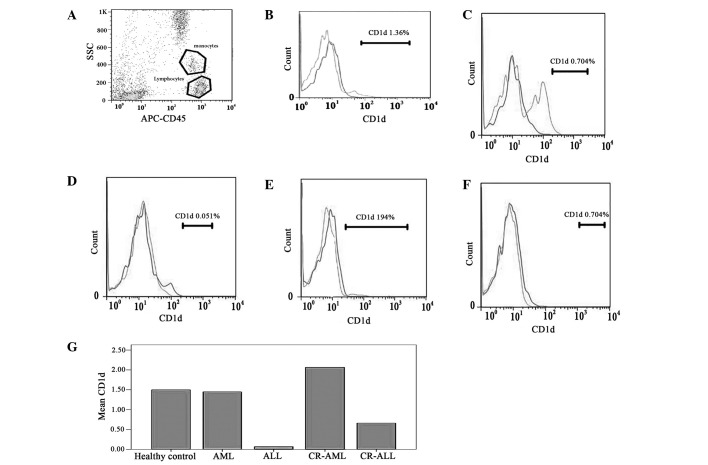
Levels of CD1d on the lymphocytes in the peripheral blood of patients with AL and CR-AL, and of healthy controls. The results showed no significant deviation between AL and AL-CR patients, and the healthy controls. (A) Monocytes and lymphocytes; (B) healthy control, (C) AML, (D) ALL, (E) CR-AML and (F) CR-ALL groups; and (G) the levels of CD1d in the different groups. AL, acute leukemia; CR, complete remission; AML, acute myeloid leukemia; ALL, acute lymphoblastic leukemia; APC, antigen-presenting cell; SCC, side scatter.

The levels of CD1d on monocytes were assessed in the PB of 56 primary AL and 28 CR-AL patients, as well as 20 healthy volunteers. A significant decrease was identified in the levels of CD1d on monocytes in the PB of AL patients (AML and ALL) compared with the healthy controls (P<0.05; Table II and Fig. 1B–D and G). Simultaneously, a difference was observed between the levels of CD1d on monocytes in CR-AML and CR-ALL patients. The levels of CD1d on monocytes remained lower in CR-AML patients than in the healthy controls (P<0.0.5; Table II and Fig. 1E and G), while the levels of CD1d on monocytes recovered in the CR-ALL patients (P>0.0.5; Table II and Fig. 1F and G). However, no difference was observed in the changing levels of CD1d on monocytes in AML and ALL patients with high (>10×10^9^/l) WBC counts (P>0.0.5; Table III). The levels of CD1d on lymphocytes were then assessed in the PB of primary AL and CR-AL patients, as well as the healthy volunteers, but no significant difference was identified (P>0.0.5, Table IV, Fig. 2). In addition, no significant difference was observed in the levels of CD1d on lymphocytes in AML and ALL patients with high (>10×10^9^/l) WBC counts (P>0.05; data not shown).

### Correlation between the number of CD3^+^CD56^+^ T lymphocytes and levels of CD1d on monocytes

In our previous study, the number and function of CD3^+^CD56^+^ T lymphocytes were found to change in AL patients (35). In the present study, the function of CD3^+^CD56^+^ T lymphocytes was decreased in AL patients (Tables V and VI). Therefore, the current study analyzed the correlation between the number of CD3^+^CD56^+^ T lymphocytes and levels of CD1d on monocytes. The results showed that the number of CD3^+^CD56^+^ T lymphocytes was increased in primary AL patients (AML and ALL patients), while the levels of CD1d on monocytes were decreased in primary AL patients. Therefore, a negative correlation was identified between the number of CD3^+^CD56^+^ T lymphocytes and the levels of CD1d on monocytes (P>0.05; Table VI). When the AL patients achieved CR, the number of CD3^+^CD56^+^ T lymphocytes returned to normal; however, the levels of CD1d exhibited two types of change. In AML patients who had achieved CR, the levels of CD1d remained lower than that in the healthy controls. Whereas in ALL patients who had achieved CR, the levels of CD1d recovered. Therefore, in ALL-CR patients, a negative correlation was observed between the number of CD3^+^CD56^+^ T lymphocytes and the levels of CD1d on monocytes (P<0.05; Table VI). The correlation between CD3^+^CD56^+^ T lymphocyte function and the levels of CD1d on monocytes was also analyzed. The results showed that the function of CD3^+^CD56^+^ T lymphocytes and the levels of CD1d on monocytes were decreased in the primary AL patients, showing a positive correlation (P<0.05; Table VII). In AL patients who had achieved CR, the function of the CD3^+^CD56^+^ T lymphocytes remained lower than that of healthy controls. Although the levels of CD1d were significantly different between the AML and ALL patients, no significant difference was identified between CD3^+^CD56^+^ T lymphocyte cytotoxicity and the levels of CD1d (P>0.05; Table VII).

## Discussion

The antitumor effect of NKT cells has been reported in several studies (10,11) analyzing the expression of CD1d on APCs (22–30,36). The results of the current study demonstrated that the levels of CD1d on monocytes were decreased in AML and ALL patients compared with healthy controls. These results also showed that the deficient antigen presentation in AML and ALL patients may be one of the reasons for AML and ALL progression. According to our previous study, a negative correlation exists between the number of CD3^+^CD56^+^ T lymphocytes and the levels of CD1d on monocytes (35). This indicated that the reason for the increase in the number of CD3^+^CD56^+^ T lymphocytes may be compensation for the deficient antigen presentation, similar to the increasing number of erythrocytes observed in patients with anoxia. However, the compensation of an increase in the number of CD3^+^CD56^+^ T lymphocytes does not prevent disease progression due to the lack of cytotoxicity of CD3^+^CD56^+^ T lymphocytes. According to other studies, T cells reactive against self-peptides are in an ignorant state known as peripheral tolerance and require activation by professional APCs in a process termed cross-priming to exert their effector functions (37–40), particularly the CD1d on APCs, which affect the function of NKT cells (41,42). Therefore, the aim of the present study was to investigate the correlation between the levels of CD1d on monocytes and the cytotoxicity of CD3^+^CD56^+^ T lymphocytes in AML and ALL patients. The results identified a positive correlation between the cytotoxicity of CD3^+^CD56^+^ T lymphocytes and the levels of CD1d on monocytes in AML and ALL patients. Therefore, the low levels of CD1d on monocytes may cause the lack of cytotoxicity of CD3^+^CD56^+^ T lymphocytes. In addition, the results showed no significant deviation between the levels of CD1d on monocytes in the AML and ALL patients with varying WBC counts. However, according to the results of our previous (35) and current study comparing the number of CD3^+^CD56^+^ T lymphocytes with varying WBC counts, no explanation has been reached concerning the compensated CD3^+^CD56^+^ T lymphocyte number in AML-1 and ALL-2 patients only. Therefore, further studies with larger sample sizes are required to clarify the clinical significance of these findings.

When AML and ALL patients achieved CR by chemotherapy, the levels of CD1d differed between the AML and ALL patients. In CR-AML patients, the levels of CD1d increased a little, but remained lower than those of the healthy controls, while levels returned to normal in CR-ALL patients. This was similar to the change in the number CD3^+^CD56^+^ T lymphocytes in CR-ALL patients. A previous review of ALL showed that young ALL patients (particularly children) may exhibit a good prognosis without SCT in certain conditions (43). As a result, the normalization of the levels of CD1d on monocytes in ALL patients may be one of the reasons for the good prognosis. However, a study by Fais *et al* (44) showed that the CD1d expression on B-precursor acute lymphoblastic leukemia subsets has poor prognosis. Further studies with the follow-up of patients who exhibit the normalization of the levels of CD1d on monocytes are required to clarify the prognosis. Our previous study showed that the levels of perforin remained low in the CD3^+^CD56^+^ T lymphocytes of CR-AML and -ALL, however, no significant correlation was identified between the levels of CD1d and perforin (35). Therefore, we consider the levels of CD1d on monocytes and the lack of cytotoxicity to be two independent prognosis factors for AML and ALL patients who have received chemotherapy.

By reviewing the literature, it was found that the levels of CD1d may also be expressed on the surface of lymphocytes (14,15). As a result, the current study tested the levels of CD1d on lymphocytes to investigate whether there was a difference between AL patients and healthy controls. However, no significant deviation was observed between the AL patients and healthy controls and, therefore, we do not consider the levels of CD1d on lymphocytes to influence the disease progression.

In conclusion, the decreasing levels of CD1d on monocytes may contribute to AML and ALL progression, as a correlation was observed between the levels of CD1d on monocytes and the number/cytotoxicity of CD3^+^CD56^+^ T lymphocytes in AML and ALL patients. Furthermore, a correlation may exist between the normalization of the levels of CD1d on monocytes in AML and ALL patients and disease prognosis. These findings suggest that the reinforcement or repair of APCs for immunocytes with antitumor effects may prolong survival in AL patients unable to undergo SCT, and may thus represent a useful strategy for treating AL.

## Figures and Tables

**Table I:** Characteristics of AML/ALL and CR-AML/-ALL patients.

Patients	n
AML	32
WBC, >10×10^9^/l	18
WBC, <10×10^9^/l	14
ALL	24
WBC, >10×10^9^/l	10
WBC, <10×10^9^/l	14
CR-AML^a^	14
CR-ALL^a^	14

a WBC count not shown as were normal.

CR, complete remission; AML, acute myeloid leukemia; ALL, acute lymphocytic leukemia; WBC, white blood cell.

**Table II:** Levels of CD1d on the monoctyes in the peripheral blood of patients with AML, ALL, CR-AML, CR-ALL and healthy controls.

Patients	CD1d, %	P-value^a^
Healthy controls	70.63±18.07	<0.05
AML	10.96±3.36	<0.05
ALL	1.21±0.57	<0.05
CR-AML	26.50±4.81	<0.05
CR-ALL	62.03±16.57	>0.05

a One-way analysis of variance.

CR, complete remission; AML, acute myeloid leukemia; ALL, acute lymphocytic leukemia.

**Table III:** Levels of CD1d on the monoctyes in the peripheral blood of patients with AML and ALL in relation to the WBC count at diagnosis.

Patients	CD1d, %	P-value^a^
AML-1	17.63±8.69	>0.05
AML-2	2.62±1.69	>0.05
ALL-1	2.49±0.47	>0.05
ALL-2	0.62±0.36	>0.05

a One-way analysis of variance.

AML-1, AML patients with increased (>10×10^9^/l) WBC counts at the time of diagnosis; ALL-1, ALL patients with increased (>10×10^9^/l) WBC counts at the time of diagnosis; AML-2, AML patients with low (<10×10^9^/l) WBC counts at diagnosis; and ALL-2, ALL patients with low (<10×10^9^/l) WBC counts at diagnosis. AML, acute myeloid leukemia; ALL, acute lymphocytic leukemia; WBC, white blood cell.

**Table IV:** Levels of CD1d on the lymphocytes in the peripheral blood of patients with AML, ALL, CR-AML, CR-ALL and healthy controls.

Patients	CD1d, %	P-value^a^
Healthy controls	1.50±0.25	>0.05
AML	1.44±0.69	>0.05
ALL	0.07±0.04	>0.05
CR-AML	2.06±0.77	>0.05
CR-ALL	0.65±0.61	>0.05

a One-way analysis of variance.

CR, complete remission; AML, acute myeloid leukemia; ALL, acute lymphocytic leukemia.

**Table V:** Number of CD3^+^CD56^+^ T lymphocytes in the peripheral blood in patients with AML, ALL, CR-AML, CR-ALL and healthy controls.

CD3^+^CD56^+^ T lymphocytes	Healthy controls	AML	ALL	CR-ALL	CR-ALL
Proportion (%)	2.72±1.58	6.05±1.83^a^	7.08±3.70^a^	3.58±1.01	3.26±1.53
Number (x10^6^/l)	58.9±34.7	162.4±54.1^a^	183.3±91.7^a^	52.4±14.4	43.5±3.9

a P<0.05 by one-way analysis of variance.

CR, complete remission; AML, acute myeloid leukemia; ALL, acute lymphoblastic leukemia.

**Table VI:** Correlation between the levels of CD1d and number of CD3^+^CD56^+^ T lymphocytes in the peripheral blood of patients with AML, ALL, CR-AML and CR-ALL.

CD1d correlation coefficient P-value		
AML	−0.278	0.041^a^
ALL	−0.273	0.048^a^
CR-AML	−0.021	0.881
CR-ALL	−0.313	0.049^a^

a Significant correlation.

CR, complete remission; AML, acute myeloid leukemia; ALL, acute lymphoblastic leukemia.

**Table VII:** Correlation between the levels CD1d and perforin on the CD3^+^CD56^+^ T lymphocytes in the peripheral blood of patients with AML, ALL, CR-AML and CR-ALL.

Patients	CD1d, %	P-value
AML	0.685	0.000^a^
ALL	0.627	0.001^a^
CR-AML	−0.171	0.220
CR-ALL	0.001	0.500

a Significant correlation.

CR, complete remission; AML, acute myeloid leukemia; ALL, acute lymphoblastic leukemia.
